# ASO Author Reflections: Disability and Outcomes Following Gastrointestinal Cancer Surgery

**DOI:** 10.1245/s10434-025-16967-w

**Published:** 2025-02-03

**Authors:** Shahzaib Zindani, Timothy M. Pawlik

**Affiliations:** Department of Surgery, The Ohio State Wexner Medical Center, James Comprehensive Cancer Center, Columbus, OH USA

## Past

Individuals with disabilities represent a substantial and underserved population in the United States, facing unique challenges within the healthcare system.^1^ Physical and mental impairments, combined with insufficiently accessible healthcare facilities, contribute to reduced engagement with preventive services, including cancer screenings.^2^ For example, women with disabilities are significantly less likely to undergo breast cancer screenings, highlighting a broader issue of healthcare inequity.^2^ Additionally, socioeconomic barriers, such as lower employment and education levels, further limit access to necessary healthcare services.^1^ Patients with disabilities are often at increased risk of health outcomes, including higher rates of comorbidities and less favorable surgical outcomes.^1,3^ While linked to adverse outcomes in chronic conditions and emergency surgeries, the specific impact of disability on complex cancer care remains poorly understood.^4^ Therefore, we sought to evaluate postoperative outcomes among patients with disabilities undergoing gastrointestinal cancer surgery and to identify the effect of disabilities on outcomes following a surgical procedure for a malignant indication.

## Present

Among 72,452 patients who underwent surgery for primary colorectal, pancreas, or hepatobiliary cancer, 7.2% of patients were affected by at least one form of disability, such as motor, visual, hearing, or intellectual. Postoperatively, patients with a disability were more likely to develop complications (4.6% vs. 3.3%), experience extended length of stay (16.3% vs. 12.0%), be discharged to a skilled nursing facility (26.6% vs. 12.3%), and have a readmission (20.0% vs. 13.5%). Patients with a disability also incurred higher expenditures in the month following surgery (disability: $24,768 [interquartile range (IQR) 17,318–38,497] vs. no disability: $20,631 [IQR 15,534–32,006]). Notably, patients with disabilities had nearly twice the odds of spending fewer healthy days at home (HDAH) versus individuals without disabilities (ref: no disability; odds ratio [OR] 1.88, 95% confidence interval [CI] 1.77–1.99). Moreover, multivariable analysis demonstrated that disability was independently associated with increased odds of complications (OR 1.36, 95% CI 1.19–1.56) and hospital readmissions (OR 1.55, 95% CI 1.44–1.66). These associations were consistent across individual disabilities as odds of experiencing low HDAH remained high in both patients with visual (OR 1.28, 95% CI 1.16–1.42) and motor impairment (2.61, 95% CI 2.41–2.82).

## Future

Surgical patients, particularly individuals with malignancy, often have multiple comorbidities and require complex care.^3^ These challenges are amplified for individuals with disabilities, who may face barriers such as limited mobility, communication difficulties, and delayed access to care.^5^ Such factors can lead to higher risks of complications and worse postoperative outcomes, including reduced HDAH.^3^ For example, motor or visual impairments can hinder mobility and proper wound care, while cognitive or sensory disabilities may complicate understanding of treatment plans and consent processes.^4,5^ To address these challenges, multidisciplinary management tailored to the needs of patients with disabilities is essential. Improved communication strategies, such as providing interpreters for hearing or visually impaired patients, and enhancing access to rehabilitation services to support mobility and recovery are needed.^5^ Employing innovative approaches to prioritize the needs of high-risk patients can help to mitigate disparities and improve overall quality of life following surgery.

## References

[1] Krahn GL, Walker DK, Correa-De-Araujo R. Persons with disabilities as an unrecognized health disparity population. *Am J Public Health*. 2015;105(S2):S198-206. 10.2105/AJPH.2014.302182. 25689212 10.2105/AJPH.2014.302182 PMC4355692

[2] Khan MMM, Waqar U, Munir MM, et al. Disparities in breast cancer screening rates among adults with and without intellectual and developmental disabilities. *Ann Surg Oncol*. 2024;31(2):911–9. 10.1245/s10434-023-14425-z. 37857986 10.1245/s10434-023-14425-z

[3] Zindani S, Khalil M, Woldesenbet S, et al. Impact of disability on postoperative outcomes after gastrointestinal cancer surgery. *Ann Surg Oncol*. 2025. 10.1245/s10434-025-16904-x. 39821489 10.1245/s10434-025-16904-x PMC11976814

[4] Rafaqat W, Lagazzi E, Abiad M, et al. The overlooked factor: the impact of disability on postoperative complications after emergency general surgery procedures. *Surgery*. 2024;176(2):232–8. 10.1016/j.surg.2024.01.037. 38480052 10.1016/j.surg.2024.01.037

[5] Rotoli J, Poffenberger C, Backster A, et al. From inequity to access: Evidence-based institutional practices to enhance care for individuals with disabilities. *AEM Educ Train*. 2023. 10.1002/aet2.10871. 37383833 10.1002/aet2.10871 PMC10294210

